# ADHD Treatment in a Community Mental Health Team: Report of a Quality Improvement Project

**DOI:** 10.1192/bjo.2026.11363

**Published:** 2026-06-30

**Authors:** Peter L Cornwall, Nikolai Rumiantsev, April Pearson, Mirelle Sutcliffe

**Affiliations:** Tees, Esk and Wear Valleys NHS Trust, Middlesbrough, United Kingdom

## Abstract

**Aims:**

The number of adult patients diagnosed with ADHD has continued to rise rapidly such that mental health services have experienced increasing delays in providing treatment. We introduced a new process in our adult CMHT to address rising demand and improve initiation and shared-care monitoring of ADHD drug treatment.

**Methods:**

We reallocated staff resources to create a dedicated ADHD clinic and collected data at baseline, 3 months and 6 months on the number of patients at the different stages of the process (waiting for initiation, initiation of treatment, monitoring of treatment), allocation of keyworker, recording of diagnosis. In addition, we measured time intervals between adding to the waiting list, initiating treatment and transition to shared-care monitoring. For patients under monitoring, we recorded the compliance with NICE standards for clinical review and for physical monitoring.

**Results:**

The number of patients with a diagnosis of ADHD wanting treatment within our service increased from 267 to 327 over 6 months (representing about 0.4% of the working age population and an increase of 22% over 6 months). With the new process, the number of patients allocated to a keyworker and with a diagnosis recorded increased to 99.7% and 99.1% respectively. The number moving from waiting to initiation phase increased substantially. Consequently, the proportion waiting for initiation fell from 17.2% to 12.5%, while the proportion in the initiation phase rose from 12.7% to 20.8%. The proportion of monitoring patients offered a review remained high (>90%), but not universal. Compliance with physical health monitoring every 12 months improved substantially, but less so for every 6 months. For annual blood pressure monitoring, compliance increased from 62.0% to 90.4% and for annual weight monitoring, the corresponding increase was from 56.7% to 80.3%. Thelongest wait for initiation reduced from 330 days to 125 days, and the median initiation phase duration decreased from 92 days to 62 days. Over the same period, the number of shared-care requests accepted by GPs increased substantially.

**Conclusion:**

The overall aims of the quality improvement project have been largely met at 6 months. Key challenges included reducing waiting times for medication initiation and accelerating transition to shared care, while maintaining NICE-compliant monitoring for established patients. We prioritised reducing initiation delays and ensuring monitoring reviews at least 12 monthly. We plan to continue the project by reducing the frequency of audit and introducing reporting on outcome measures.

